# The Changing Natural History of Anisometropia: A Scoping Review

**DOI:** 10.1007/s44402-026-00029-z

**Published:** 2026-03-05

**Authors:** Bruce J. W. Evans, Rakhee Shah, Natalia Vlasak

**Affiliations:** 1 Optometry and Visual Sciences at City St George’s, University of London, London, UK; 2 HOYA Vision Care, Amsterdam, The Netherlands

**Keywords:** Amblyopia, Aniseikonia, Anisometropia, Myopia control, Refractive error, Stereopsis

## Abstract

**Purpose:**

To review literature on anisometropia, concentrating on diagnostic criteria, contemporary prevalence and progression with regard to the changing distribution of refractive errors in many countries. Also, to consider anisometropia with respect to myopia and hyperopia control, regions/race/ethnicity, effects on visual function and associated conditions.

**Methods:**

Scoping review based on searches of PubMed, Embase and Cochrane databases.

**Results:**

Various diagnostic criteria have been used for anisometropia, most commonly a SER difference ≥1.00 D. Anisometropia is more common in people with higher refractive errors, and therefore, its prevalence changes with the frequency distribution of refractive errors. Anisometropia is traditionally mostly associated with hyperopia, and this is still the case in some populations. In East and South-East Asia, the rapid increase in myopia has resulted in increased anisometropia. This is associated with impaired stereopsis and binocularity, as well as increased rates of strabismus and amblyopia. When anisometropia is corrected with spectacles, there is an increased risk of spectacle non-tolerance arising from aniseikonia (different image sizes in each eye) and prismatic effects. Contact lenses alleviate most of the problems associated with anisometropia, but are under-prescribed for this condition. The increased association between anisometropia and myopia has led to trials of myopia control interventions, which show promise for reducing anisometropia in myopic cases. However, since myopia in one eye is often a precursor of bilateral myopia, the likelihood of pre-myopia in the non-myopic eye should be considered.

**Conclusions:**

The association between anisometropia and the magnitude of refractive error means that in populations with a high prevalence of myopia, anisometropia has largely become a feature of that refractive error, in contrast to the traditional association with hyperopia. This has important implications for myopia control. Vision screening and/or routine professional eye care are recommended because anisometropia is under-diagnosed.

Key Points

- Anisometropia is increasing in prevalence in populations with increasing myopia.
- Contact lenses alleviate most of the problems associated with anisometropia.
- Myopia control interventions can be helpful for myopic anisometropia.
- Vision screening and/or routine professional eye care are recommended because anisometropia is under-diagnosed.

## Introduction

Anisometropia occurs when there is a significant difference between the refractive errors of the two eyes [1]. Antimetropia refers to anisometropia when one eye is hyperopic and the other myopic [1]. Anisometropia has been described as “the least understood refractive abnormality” [2]. It can cause symptoms (e.g., asthenopia, double vision), reduced visual performance, spectacle non-tolerance, amblyopia and strabismus [3].

Small differences in the refractive errors of the eyes (<1.00 D) are common and not usually problematic. However, Flitcroft et al. found that in young children (aged 6–7 years) in Northern Ireland, small degrees of anisometropia (≥0.50 D) are associated with impaired emmetropisation (in this population, typically hyperopia) [4]. These authors noted that both eyes of one individual share the same environment and genes and suggested that anisometropia is a marker for poorly regulated eye growth.

Hyperopic anisometropia has a stronger association with amblyopia than myopic anisometropia [5, 6], and the failure to detect and correct clinically significant anisometropia can lead to visual impairment in the anisometropic eye [7]. Parents are often unaware of anisometropia, and so vision screening [8] and/or regular optometric eye examinations of young children are important [9].

The purpose of this paper is to review the literature on anisometropia, concentrating on contemporary prevalence and progression with regard to the changing distribution of refractive errors in many countries. The review also considers anisometropia with respect to myopia and hyperopia control, diagnostic criteria, race/ethnicity/region, and the effects of anisometropia on visual function.

A scoping review [10, 11] was considered appropriate because the review aims to address broad research questions, which include a variety of evidence sources and clarify key concepts/characteristics in the literature. A systematic approach was adopted for searching the literature. The review protocol was not pre-registered, but a Preferred Reporting Items for Systematic Reviews and Meta-Analyses extension for Scoping Reviews (PRISMA-ScR) checklist was followed.

## Methods

PubMed, Embase and Cochrane databases were searched using the keywords and date limits in Table 1, last updated October 2025. In view of the changing distribution of refractive errors, especially myopia [12], the prevalence of anisometropia and its interaction with race, ethnicity and regionality is likely to change over time. Therefore, to establish contemporary prevalence, these searches were limited to the last 5 years. Concerning the link between anisometropia and visual functions, it was considered that confining the search to the last 15 years would provide an adequate review of research in this field, whilst keeping the manuscript to a manageable length. There is a much smaller literature on anisometropia and myopia control, and therefore, no date limits were required for this topic.

**Table 1:** Search terms and date limits used in the literature search.

Search terms (any field)	Limited to
Anisometrop* AND prevalence	2020–2025
Anisometrop* AND (race OR ethnicity)	2020–2025
Anisometrop* AND “visual function”	2010–2025
Anisometrop* AND (“myopia control” OR “myopia management”)	NIL

General inclusion criteria were relevant to the topic, and conclusions were supported by methods and analyses. Studies on prevalence were included if they specified the diagnostic criteria for anisometropia and evaluated a general (non-clinical) population. General exclusion criteria were non-human studies, non-English reports and publications where only the abstract was accessible, unless the abstract provided adequate information to meet the inclusion criteria, which was the case for only seven publications. The key tools used in handling and synthesising results were EndNote and Microsoft Excel.

All publications were screened by title and abstract, and duplicates were removed by authors BE or RS, with uncertain cases proceeding to full report review. Full reports were studied by BE or RS, with uncertain cases discussed by both authors, and in every case, consensus on inclusion was reached. At all stages, the selection criteria above were applied. Relevant data were extracted directly from the draft manuscript. Additional references were identified from article reference lists and the authors’ bibliographies. When describing race/ethnicity, this review uses the terminology in the articles under review.

## Results

### Literature Search

**Fig. 1:**
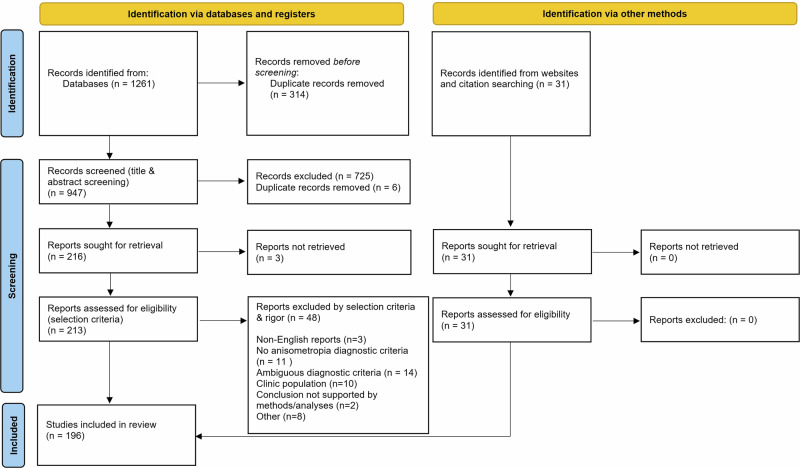
Preferred Reporting Items for Systematic Reviews and Meta-Analyses (PRISMA) flowchart of manuscript selection. The chart shows the identification, screening and inclusion stages. *n* number of reports.

Figure 1 is a PRISMA chart summarising the output of the literature search.

### Diagnostic Criteria for Anisometropia

Anisometropia is typically defined as present or absent, and prevalence is therefore dependent on the criterion used [13]. For studies included in this review that have adopted this approach, the criteria are summarised in Table 2 (studies that investigate multiple criteria appear in the table more than once). Manuscripts that do not specify the diagnostic criteria for anisometropia, including those that do not specify whether they are referring to spherical equivalent refraction (SER) or some other variable (e.g., spherical component of a sphero-cylinder prescription) [14–29], are excluded from Table 2.

**Table 2:** Definitions of anisometropia used in the literature—grouped by common thresholds, refractive sign (myopia vs. hyperopia), astigmatic criteria and vector-based definitions.

Category/Label	Anisometropic threshold	Studies	Additional comments
Studies using lower criteria than SER ≥ 1.00 D	SER ≥ 0.50 D	[183–185]	
	SER > 0.50 D	[186–188]	
Studies using SER ≥ 1.00 D	SER ≥ 1.00 D	[6, 7, 12, 33, 38, 48–50, 53–55, 57–59, 61–63, 67, 75–78, 87–90, 104, 119, 128, 133–135, 137, 148–150, 154, 155, 166–168, 172, 175, 185, 189–226]	Most commonly used criterion, used in >80 studies
Studies using higher SER thresholds	SER > 1.00 D	[51, 52, 79, 139, 153, 159, 186, 227–238]	
	SER ≥ 1.25 D	[239]	
	SER ≥ 1.50 D	[44, 124, 131, 240–245]	
	SER > 1.50 D	[145]	
	SER ≥ 2.00 D	[15, 110, 132, 138, 167, 246–250]	
	SER > 2.50 D	[56, 249]	
	SER ≥ 3.00 D	[56, 158] (myopes only) [251]	
Studies distinguishing hyperopic vs. myopic anisometropia	Hyperopes: SER ≥ 0.75–1.50 D; Myopes: SER ≥ 1.25–3.00 D	[91, 92, 127, 173, 251–257]	Usually SER-based, but thresholds vary by refractive error type (hyperopia vs. myopia)
Studies including astigmatic anisometropia	Cylindrical difference often ≥1.00–1.50 D between eyes	[6, 9, 12, 14, 23, 40, 44, 45, 60, 91, 119, 126, 127, 135, 138, 150, 172, 173, 183, 184, 194, 195, 199, 201, 205, 209, 210, 216, 239, 243, 244, 246, 251–253, 256–260]	In some studies, astigmatic anisometropia is described with SER criteria, in others with the spherical component of refractive error
Vector/specialised definitions	Vector criteria (e.g., *J*_0_/*J*_45_ ≥ 0.5); anisometropia at the most anisometropic meridian ≥1.00 D	Vector analysis (*J*_0_, *J*_45_ ≥ 0.5) [6, 33, 35] ≥1.00 D at most anisometropic meridian [94]	Uses vector analysis (power-vector) or meridian-specific criteria, which [190] integrates axis as well as magnitude

*SER* spherical equivalent refraction.

The most common approaches are to consider the SER solely or to consider both the SER and the astigmatic component of the sphero-cylinder refractive error (there is a significant correlation between spherical and astigmatic anisometropia) [30]. An unusual approach is to consider only the spherical component of the sphero-cylindrical refraction [31, 32]. A thorough approach was adopted by Borchert et al. [33], who considered both SER and cylindrical (astigmatic) anisometropia. Cylindrical anisometropia was defined as the difference in absolute cylinder between the eyes, regardless of axis. To account for interocular differences in cylinder axis, the authors also calculated the vertical Jackson cross cylinder vector (*J*_0_) and oblique Jackson cross cylinder vector (*J*_45_) for each eye using the formula of Thibos et al. [34]. Anisometropia by vector analysis has been defined as a difference of ≥0.5 in *J*_0_ or *J*_45_ between the two eyes, equivalent to a cylinder power difference of ≥1.0 D at 0° and 45°, respectively [33]. Dobson et al. compared two different vector approaches [6], while other anisometropia researchers have also used vector analysis [35, 36].

Qiao et al. classified anisometropia as low (SER ≥ 1.00 D), moderate (SER ≥ 2.00 D) or high (SER ≥ 3.00 D) [37]. Moshkovsky et al. classified anisometropia as moderate when the SER difference between the eyes was 1.00 to <3.00  D and severe when ≥3.00 D [38]. Other authors have used different classifications of mild/moderate/severe [39].

The American Academy of Paediatric Ophthalmology and Strabismus (AAPOS) Vision Screening Committee produced criteria for various amblyogenic risk factors detected by screening, which included anisometropia (spherical or cylindrical) >1.50 D [40]. These criteria are used by some screening instruments [41] and in other studies [42]. They were updated in 2013 and again in 2021 when the anisometropia criterion was reduced to >1.25 D (defined as the magnitude of the difference in the lesser meridian) [43]. Some recent studies continue to use the >1.50 D anisometropia cut-off [44, 45].

### Prevalence and Progression of Anisometropia

#### Overview

**Fig. 2:**
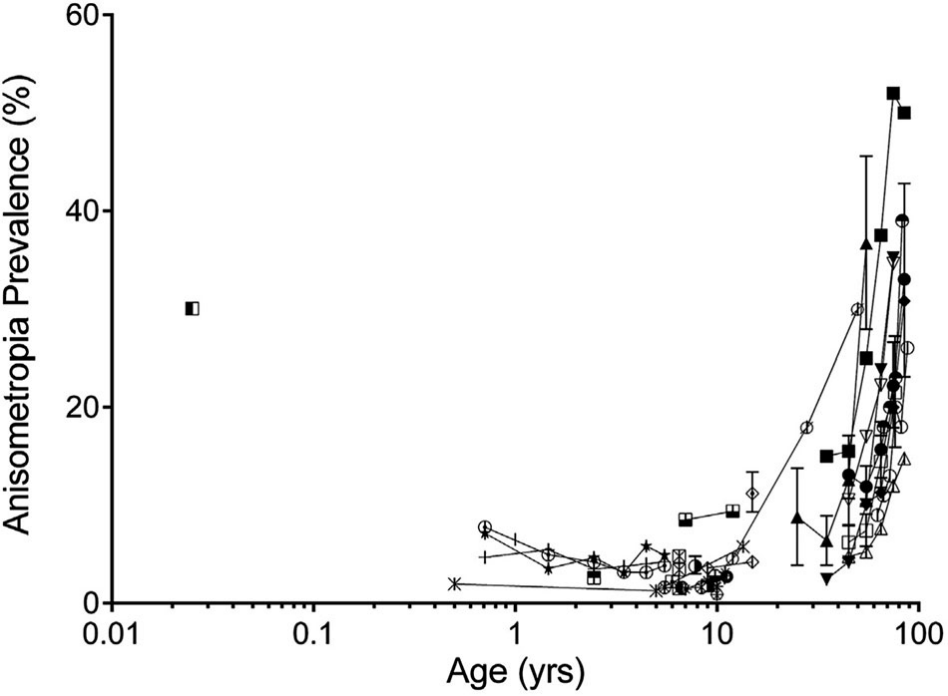
Prevalence of anisometropia as a function of age. To account for differences in the criteria used to diagnose anisometropia, only studies employing the most commonly used criterion are included, namely a difference of ≥1.00 D SER between the right and left eyes. Reproduced with permission from Barrett et al. [13]. SER spherical equivalent refraction.

Early reviews by Barrett et al. and Vincent et al. noted that anisometropia prevalence varies with age [13, 46] and is relatively high in the weeks following birth, in the teenage years, coinciding with the onset of myopia, and in older adults after the onset of presbyopia [13]. Barrett et al.’s thorough review includes Fig. 2 and describes anisometropia as stable between the ages of 20–40 years, with ~11–13% of people exhibiting anisometropia ≥1.00 D [13]. These authors note that most studies are cross-sectional, but that longitudinal studies indicate that most children who were anisometropic at one examination were not anisometropic on subsequent evaluations, but in cross-sectional studies had been replaced by children who did not exhibit anisometropia at earlier dates [13]. Anisometropia in older children was found to be more stable. Haegerstrom-Portnoy et al. studied an elderly population and considered the function describing anisometropia with age as likely exponential [36].

The review by Barrett et al. found limited evidence at that time (see “Discussion”) that gender, racial or ethnic differences exert a major influence on anisometropia prevalence [13]. These authors commented that anisometropia has about one-third the prevalence of bilateral refractive errors of the same magnitude and is higher in highly ametropic groups, suggesting that similar emmetropisation failures underlie ametropia and anisometropia.

Considering regional variations, there has been a marked change over the years in the distribution of refractive errors, with South-East and East Asian populations experiencing accelerated myopic transitions, particularly in children [47]. This is likely to influence the prevalence of anisometropia because of Barrett et al.’s observation that anisometropia prevalence increases with ametropia [13]. Therefore, Table 3 shows recent studies of anisometropia prevalence divided into populations in East and South-East Asia and from other regions.

**Table 3:** Summary of cross-sectional population-based studies since 2020 on the prevalence of anisometropia, with respect to age and authors’ description of population, divided into populations from South-East Asia and elsewhere.

Authors	Year	Age (yr)	Population	Prevalence	Comments
*Populations predominantly not from South-East and East Asia*					
Margines et al. [23]	2020	3–5	79% Latino; ~5% each for Asian, Black/African American, White, Other/Unknown	20% spherical 24% cylindrical	
Hashemi et al. [50]	2020	18–48	Iranian university students	3.6%	Prevalence of myopia 43%.
Lingham et al. [51]	2020	20	Western Australia	2.9%	Of the anisometropes, 33% had myopia and 27% hyperopia.
Nunes et al. [239]	2021	3–16	Portugal	6.1%	Prevalence is higher in older adolescents (9.4%) than in pre-school (2.9%).
Kiatos et al. [216]	2021	1.5–6	Ontario, Canada	2.8% spherical, 1.8% cylindrical	
Guo et al. [203]	2022	4–14	Mostly “Black”, some “Latinx”	31%	
Kulp et al. [173]	2022	3–5	African American, American Indian, Asian, Hispanic, non-Hispanic white	3–7%	Highest prevalence in Hispanic population and, at this age, lowest in Asian.
Guillon-Rolf et al. [242]	2022	2–12	French	5.0%	
Yasir et al. [249]	2022	See comments	Children (primary and secondary school), Saudi Arabia	3.0%	Age not given but pre-kindergarten to 12th grade. Unusually high criterion for anisometropia (>2.50 D).
Tajbakhsh et al. [219]	2022	6–12	Iran	4.0%	
Hashemi et al. [231]	2022	>60	Iran	23.8%	Anisometropia in 24.9% of males; 22.8% of females.
Monika et al. [206]	2023	8	Polish	4.0%	
Ramirez-Ortiz et al. [215]	2023	6–12	Mexican	≥1.00 D: 7.9% > 1.50 D: 3.9%	Anisometropia increases with age.
Rabiu et al. [208]	2023	≥40	Emiratis Non-Emiratis	11.4% 9.2%	Myopia prevalence higher in Emiratis.
Hashemi et al. [60]	2024	6–12	Iranian	1.1%	Anisometropia stable aged 6–12 years; highest prevalence in myopes.
Nguyen et al. [211]	2024	See comments	USA	28.5%	Age not given, pre-kindergarten to 12th grade. Prevalence lower in 3rd–4th Grade than pre-school.
Pang et al. [54]	2024	2–15	USA—Black –Hispanic	14% 8%	
Behboudi et al. [220]	2024	≥50	Iran	16.9%	
Li et al. [19]	2025	4–7	Australian	13.7%	
Nejatian et al. (meta-analysis) [221]	2025	children	Australian—Indigenous –non-Indigenous	4.1% 5.0%	
Moshkovsky et al. [38]	2025	1–6	Israel	6.6%	
Voigt et al. [55]	2025	35–74	German	14.5%	
Findlay et al. [257]	2025	7–10	Aotearoa, New Zealand	3%	
*Populations predominantly from South-East and East Asia*					
Cheng et al. [52]	2021	≥30	Rural China	18.8%	Two-thirds of the population were myopic.
Xu et al. [205]	2022	4–17	Chinese	13.2%	
Wang et al. [204]	2022	6–18	Chinese	6.8%	Urban habitation, higher household income and increasing age were risk factors for anisometropia.
Lillvis et al. [217]	2022	≥40	Timor-Leste	17.8%	
Shi et al. [218]	2022	6–23	China	4.8–14.8%	Varying rates in five ethnic groups. Anisometropia increased with age, slowing in later childhood.
Challa et al. [244]	2022	See comments	Chinese	8.8%	Age not given but kindergarten to senior school.
Lin et al. [159]	2023	≥30	Chinese adults with type 2 diabetes	17.2%	
Wang et al. [59]	2023	6–10	Hong Kong Chinese	6.4–17.0%	
Zhou et al. [207]	2023	7–19	Chinese	25.6%	Anisometropia prevalence increased from 7 years (7.8%) to 19 years (39%) old, mirroring increasing myopia.
Chen et al. [170]	2024	12–18	Chinese	32.8%	No relationship between anisometropia and age.
Fang et al. [228]	2024	6–18	Chinese—Urban–Rural	21.3–22.0%	Decreased time outdoors and increased near vision associated with higher risk of myopia and anisometropia.
Miki et al. [229]	2024	20–90	Japan	14.9%	
Yao et al. [213]	2024	6–12	Chinese	16.5%	Anisometropia severity increases with age. Most participants were myopic.
Zhou et al. [175]	2024	12–19	Chinese	33%	
Sun et al. [212]	2025	2–8	Chinese	1.8%	
Zhou et al. [222]	2025	7–19	Chinese	25.6%	Anisometropia prevalence increased with age.
Xiao et al. [128]	2025	3–7	Chinese	18.2%	

The studies employed a variety of methods (e.g., differing in whether refractive error assessment included cycloplegia and in diagnostic criteria, detailed in Table 2).

Differences in age of population and in the criteria for anisometropia explain some of the variability in Table 3. Another source of variation is that not all studies used cycloplegic refraction. Studies that have investigated longitudinal trends are considered below. One prevalence study that is not included in Table 3 because it is of a clinical population of refractive surgery candidates is noteworthy because it demonstrated that the relationship between anisometropia and age was positive in myopes and negative in hyperopes [48]. In a similar study of refractive surgery candidates in China, Wang et al. found a U-shaped curve in the correlation of myopic anisometropia and SER: anisometropia tended to be greater in individuals with the highest and lowest degrees of myopia [49].

Considering the prevalence rates for spherical anisometropia in Table 3, the mean prevalence for East and South-East Asian populations is 18.3%, compared with 10.3% for other populations. However, this does not take account of differing age ranges and diagnostic criteria. Just considering studies of populations aged ~20–40 years (when Barrett et al. stated that anisometropia is stable) and those that used comparable criteria, the mean of the two studies not from East or South-East Asian populations [50, 51] is 3.25%, compared with 18.8% in a comparable study in rural China [52]. Another investigation of a similar age range and criterion in China that only considered myopes found a prevalence of anisometropia of 30% [49]. Therefore, the following review of recent longitudinal studies of anisometropia is divided into populations from East and South-East Asia and from other regions.

#### Longitudinal Studies of Populations Predominantly from Regions not in East and South-East Asia

In a longitudinal study of the influence of prenatal environment in a Western Australian population, an increased risk of anisometropia at age 20 years was associated with older than usual maternal age and an abnormal umbilical cord at birth [51]. In Israel, Kinori et al. undertook a longitudinal study of 33,496 children aged 1–6 years who did not have anisometropia at baseline [53]. At baseline, 69.1% were hyperopic, 26.7% emmetropic and 4.2% myopic. After a mean interval of 5 years, 7.7% were diagnosed with anisometropia. Adjusted odds ratios for anisometropia gradually increased with baseline refractive error severity, reaching 13.90 (5.32–36.34) in severe myopia and 4.19 (3.42–5.15) in severe hyperopia.

In a large USA longitudinal study, Pang et al., found the prevalence of anisometropia was higher in Black than Hispanic children, but the prevalence of anisometropia did not change with age [54]. In a longitudinal study of children aged 1–6 years in Israel, Moshkovsky et al. selected children with anisometropia and followed them for an average of 5.1 years (SD 2.4) [38]. They found that higher degrees of baseline anisometropia, hyperopia, myopia and astigmatism were associated with increased prevalence of future anisometropia. Voigt et al. in Germany similarly found that with increasing myopia and hyperopia, there was a V-shaped increase in anisometropia with minimal anisometropia at emmetropia [55].

#### Longitudinal Studies of Populations Predominantly from East and South-East Asia

Shih et al. reported a longitudinal study from Taiwan, concentrating on children under the age of 10 years who had visited a clinic because of monocular amblyopia and were found to have anisometropia of at least 3.00 D [56]. Children were followed up for a minimum of 2 years. In myopic anisometropes, myopia in the better eye increased more rapidly than that of the more myopic eye, which resulted in a yearly reduction of anisometropia of 0.51 D. In the hyperopic anisometropes, hyperopia decreased synchronously in both eyes [56].

Lee et al., in a 2-year longitudinal study of 7035 primary school children in Taipei City, studied children who were selected as having no anisometropia at baseline (therefore excluded from Table 3) [57]. The average annual incidence of myopia was 3.8%. Over 2 years, the proportions of hyperopes, emmetropes and myopes who developed anisometropia were 4.9%, 6.7% and 10.6%, respectively. For hyperopic children, the baseline refractive error and having one myopic parent were significantly associated with an increased risk of anisometropia. For emmetropic children, baseline refractive error, female sex, performing near work at a distance of <30 cm, and reading while lying on a bed were significant risk factors. Lee et al. noted that in Taiwan, the prevalence of myopia reaches ~80–90%, so the results may not be generalisable to other, less myopic populations. In this population, childhood anisometropia results from a faster rate of myopia progression in the more myopic eye from baseline.

In another 2-year longitudinal study from Taiwan, Lin et al. found that anisometropia decreased in children aged <6 years and increased in older children [58]. In children aged 3–6 years, the mean anisometropia was higher in children with myopia and antimetropia than in those with hyperopia. The differences were not statistically significant in children aged >6 years. Even at the first visit, when children were 5–6 years old, approximately half the population were myopic in at least one eye. Despite the association with myopia, high anisometropia was associated with amblyopia. Unlike Western populations, when strabismus in anisometropic individuals tends to be esotropia [3], in the largely myopic Taiwanese children, strabismus tended to be exotropia.

In a longitudinal study of Hong Kong Chinese children, Wang et al. found an increase in anisometropia between the ages of 6–10 years, associated with imbalanced axial elongation [59]. These changes were found to be linked with two genetic factors that are associated with scleral and myopic changes. The mean refractive error by the age of 10 years was myopic.

In a retrospective study, Wang et al. investigated anisometropia resulting from unilateral myopia in Chinese children [18]. In children who had not been prescribed spectacles to correct the unilateral myopia, the myopic eye progressed more rapidly than the fellow eye, but in cases wearing spectacles, the two eyes progressed at a similar rate.

### Myopia Control and Hyperopia Control Interventions to Reduce Anisometropia

Axial length is the most important biometric factor in anisometropia [60], and in children with hyperopic anisometropia, axial elongation is slower in the more hyperopic than in the less hyperopic eye [61]. A study of Chinese children using ultra-widefield swept-source optical coherence tomography found that anisometropic children exhibit macular steepening and peripheral flattening in the more myopic eye during axial elongation [62]. Studies of Chinese children [63] and young adults [64] with myopic anisometropia found that the more myopic eyes showed increased peripheral hyperopic defocus.

A limitation of many clinical trials of myopia control interventions is that they exclude participants with significant degrees of anisometropia, limiting the evidence base for managing these patients [65, 66].

For anisometropic children who have one myopic eye and the other eye non-myopic, one management option is to undertake orthokeratology (OK) just in the myopic eye. Several studies have found that monocular OK of the myopic eye slows axial elongation of this eye and, over time, reduces anisometropia [67–74].

For patients with anisometropic bilateral myopia who are fitted with OK lenses in both eyes, most studies have found that OK is more effective at slowing progression in the more myopic eye than in the less myopic eye, thus reducing anisometropia over time [67, 75–83]. Only two studies (with sample sizes of 25 and 47) found that OK, although effective at slowing axial elongation in both eyes, did not reduce anisometropia [70, 72].

Zhai et al. investigated the effect of fitting Chinese children having unilateral myopia with highly aspheric lenslet (HAL) spectacle lenses [73]. The axial elongation of both eyes was slowed by a similar amount. This study also included a group who received OK to the myopic eye only. The results show that the children who did not receive a myopia control intervention in the non-myopic eye tended to have rapid axial elongation in this eye. In other words, unilateral myopia heralds myopic progression in the better eye.

**Fig. 3:**
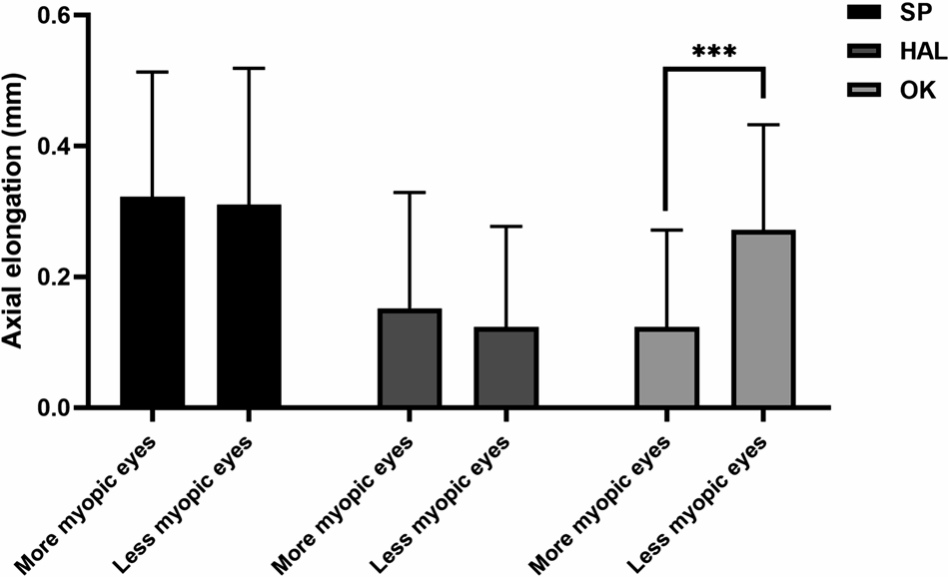
For bilateral myopic anisometropia, comparison of axial elongation in response to wearing lenses between the more and less myopic eyes with three methods of bilateral myopia control. HAL highly aspherical lenslet spectacles, OK orthokeratology, SP single vision spectacles; ***p < 0.001. Reproduced under Creative Commons from Chen M, Yang X, Yu X, Lei H, Mao X. Comparing the effects of OK lenses and highly aspherical lenslets on axial length in myopic anisometropia. BMC Ophthalmol 2025;25:312 [68].

In a retrospective study of Chinese children with bilateral myopia and anisometropia, Chen et al. compared cases who were prescribed either single vision spectacles (SP), HAL spectacles or OK to both eyes [67]. As Fig. 3 shows, over 1 year, the children wearing SP demonstrated similar axial elongation in each eye. HAL lenses were associated with less axial elongation, but to a similar degree in each eye, so that anisometropia did not increase. In contrast, in the OK group, axial elongation slowed more in the eye with higher myopia, reducing axial elongation. The underlying mechanism for this finding is considered below in the “Discussion” section.

Concerning myopia control with atropine [84], a 1% concentration prescribed to the more myopic eye has been found to reduce anisometropia [85], as have 0.125% [86] and 0.025–0.05% concentrations [22]. In contrast, there was no [21] or a minimal [87] treatment effect on anisometropia for 0.01% atropine.

Wang et al. used low-level red-light (RLRL) treatment on only the more myopic eye of children with anisometropic myopia [88]. The treatment was effective at reducing myopia progression and axial elongation of the treated eye, therefore reducing anisometropia.

Beasley et al. investigated whether multifocal contact lenses with a centre-near design fitted to the more hyperopic eye could reduce anisometropic hyperopia [89]. After 2 years of wear, there was no significant treatment effect with regard to inducing axial elongation or correcting anisometropia.

### The Effect of Anisometropia on Visual Functions

#### Amblyopia

Anisometropia is a major risk factor for amblyopia [9, 35, 37, 90–93]. Hyperopic anisometropia has a stronger association with amblyopia than myopic anisometropia [5, 6], with the risk of amblyopia approximately twice as great in hyperopic as in myopic anisometropes of comparable refractive imbalance [94]. For example, for ~3 D of anisometropia, ~40% of hyperopic anisometropes are amblyopic compared with ~15% of myopic anisometropes [94]. In a longitudinal study in children aged 4–12 years with hyperopic anisometropia in Texas, USA, the children with amblyopia had a greater increase in the magnitude of anisometropia over time than those without amblyopia [61]. Anisometropic amblyopia has been thoroughly reviewed elsewhere [3, 13, 61, 95] and will not be considered further in the present review.

An approach for treating amblyopia is to prescribe atropine eye drops in the unaffected eye [3]. Such cases may benefit from wearing bifocals, which Tejedor et al. showed can be tolerated by anisometropic children using atropine [96].

#### Aniseikonia

Aniseikonia is a perceived difference in size and/or shape of the visual images of the two eyes [1]. It can result from inequalities in axial lengths, retinal elements or cortical representation of the monocular images. Aniseikonia is induced by spectacle lenses of different powers used in the correction of anisometropia [1].

A hypothesis, Knapp’s law [1], predicts that for axial anisometropia the difference in relative spectacle magnification will be minimised by wearing spectacles, not contact lenses. However, this assumes that the retinal receptors are equally spaced in each eye in anisometropia, which, since the retinal area would differ in each eye, seems unlikely [97, 98]. An experimental investigation by Winn et al. disproved Knapp’s law by demonstrating that contact lens correction minimises aniseikonia in axial as well as refractive anisometropia [99]. They concluded that in young patients, contact lenses provide the most potent binocular stimulus to the visual system [100]. Other research supports Winn et al.’s findings [98] and shows that this is explained by photoreceptor spacing [101].

A review by South et al. noted that both anisometropia itself and the optical correction of anisometropia can cause aniseikonia [102]. They noted that aniseikonia may not be experienced by the patient under normal binocular viewing conditions if the image from the amblyopic eye is of poor quality, strongly suppressed or if there are cortical adaptations.

“Rules of thumb” are often applied to aniseikonia, some of which date back 75 years [103]. One such recommendation is the amount of aniseikonia that is induced per 1 D of anisometropia in spectacle correction, with typical values given as 1% [102, 104–106], 1.5% [107] or 1–1.5% [103, 108, 109]. Using a sensitive measure of aniseikonia, Takigawa et al. found that the amount of aniseikonia per dioptre was 1.1% and per mm of aniso-axial length was 3.1–3.4% [110]. Stokkerman and Day caution that these rules of thumb significantly overestimate aniseikonia, and direct measurements are more accurate [108]. In children aged 3 months–12 years, Bharadway and Candy showed that accommodative and vergence gains are remarkably robust to up to 4 D of lens-induced anisometropia and 11% for induced aniseikonia [111]. Nonetheless, these authors noted that the impact of anisometropia in young children is likely to include disruptions of image correspondence and accommodative performance.

Another rule of thumb is the limit of anisometropia or aniseikonia that can be tolerated without symptoms. For anisometropia, this has been cited as 2.00 D [104] or 3.00 D [104, 112]. For aniseikonia, the maximum that can be tolerated is given as 2% or 5% [3]. This means that anisometropia of as little as 1.25 D may cause clinically significant aniseikonia, although the precise value will depend on the prescription, back vertex distance and relative ocular dimensions [113].

Krarup et al. carried out a retrospective observational study of patients with anisometropia induced by cataract surgery [104]. Aniseikonia tolerance range (ATR) was defined as the total amount of optical aniseikonia (measured with afocal size lenses that provide magnification but no dioptric power [1]) before stereoacuity is impaired. Of the 123 cases, 26 described symptoms after surgery that disappeared within a week to a month. Regression analysis demonstrated a slope of 0.04% aniseikonia per dioptre of anisometropia (95% CI: −0.02 to 0.12). Commenting on the lack of a significant correlation between anisometropia and aniseikonia instead of the ~1:1 relationship expected by the rule of thumb, the authors cite previous work supporting their finding, which was likely due to adaptation in the visual system.

The findings of Krarup et al. were to some extent predicted by an experiment by Burian in 1943 [114]. He found that when a person with normal binocular vision wears a meridional size lens placed at axis 90 in front of one eye for several days, they initially experience a typical distortion of the surroundings. The longer the lens is worn, the less the distortion and it finally disappears and is absent as long as the observer remains in normal visual environments. However, if the number of effective perspective factors in the surroundings is negligible, the distortion recurs immediately, no matter how long the size lens is worn. Burian noted that fatigue could reduce the effect of adaptation and found individual differences in the ability to adapt [114]. Limitations of this work are that only three observers were studied, over a period of 8–14 days.

In further research on the ATR in 32 normal patients over the age of 50 years, Krarup et al. found large inter-individual differences in ATR: 19%, 3%, 22% and 54% of participants had an ATR of ≤1%, 1–5%, 5–10% and >10%, respectively [115]. Other research also found large inter-individual variation in the amount of aniseikonia that can be tolerated [116]. For some individuals, symptoms (e.g., headache, intermittent diplopia and spatial distortions) can lead to non-tolerance of new spectacles [3] and reduction in stereoacuity [117].

The investigation and management of aniseikonia are beyond the scope of the present review, but are covered elsewhere [3, 100].

#### Differential Prismatic Effects

Prismatic effects are induced when the eyes look away from the optical centres of spectacle lenses. When two spectacle lenses have different powers, the eyes need to move by different amounts and this can be problematic, especially for vertical eye movements required with multifocal lenses [3]. The investigation and management of differential prismatic effects are considered in detail elsewhere [3, 113]. Contact lenses [100] or refractive surgery provide solutions to differential prismatic effects [118].

#### Stereopsis

Dobson et al. found that compared with best-corrected visual acuity, best-corrected random dot stereoacuity is adversely affected by lower degrees of anisometropia (≥0.50 D) [6]. Unlike visual acuity, stereoacuity was equally impaired by low degrees of myopic and hyperopic (and astigmatic) anisometropia. Lee et al. found no significant relationship between the severity of anisometropia and the level of stereopsis [119]. However, the stereoacuity test had a ceiling effect, only testing to 40 s of arc [3].

Gawecki found that myopic anisometropia >2.00 D can cause significant impairment of stereoacuity, especially for distance vision and against-the-rule astigmatism [120]. A limitation of the study and others that induce anisometropia [111, 121–123] is that short-term induced anisometropia may not predict the effect of permanent anisometropia in a developing child. Other studies have found that simulated anisometropia is associated with loss of clarity, increased binocular difficulties, reduced stereoacuity and visual discomfort [122], as well as impaired academic-related performance [123].

The real-world impact of reduced stereoacuity from anisometropia was highlighted by Kiziltan Eliacik and Eliacik [124], who hypothesised that reduced stereoacuity from anisometropia may lead to traumatic dental injuries (TDI). They compared 75 children with TDI to 65 age-matched controls with good dental health. Abnormal stereoacuity was present in 28% of the TDI group compared with 3% of controls, and increasing anisometropia was correlated with worse stereopsis.

Atchison et al. studied normal observers to investigate whether aniseikonia induced by size lenses could be neutralised by equal and opposite screen-induced aniseikonia, created by computer software [125]. The researchers were not able to neutralise lens-induced aniseikonia fully, possibly because lens-induced effects involve the whole visual field.

In a cross-sectional study, Elamurugan et al. investigated the association between different types of refractive error and stereoacuity in children having a mean age of 13 years [126]. Spherical refractive errors had the least impact on stereoacuity, with astigmatism having a greater effect and anisometropia the greatest effect. Chen demonstrated that higher degrees of anisometropia have significantly better stereoacuity following OK compared with spectacles [39].

Hashemi et al. tested 5620 Iranian children aged 6–12 years [127]. Three years later, they retested 4666 of the children from the initial sample and used multiple linear regression to investigate which optometric parameters at the first appointment were predictive of a reduction in stereoacuity after 3 years. Spherical, but not astigmatic anisometropia, was predictive of a reduction of stereoacuity. Other studies have confirmed the relationship between anisometropia and reduced stereoacuity [37, 60, 124, 128, 129] and that the most disruptive form of anisometropia is spherical hyperopic anisometropia [44].

Wang et al. [112] compared two optometric approaches for prescribing prisms [3], the measuring and correction methods of Haase (MCH) and the Optometric Extension Programme. The study was not a randomised controlled trial and only evaluated the immediate effect of spectacle corrections with a trial frame, but found significantly better stereoacuity and binocular visual acuity with the MCH approach. The Mallett approach, commonly used in the UK [3], was not investigated.

#### Impact of Anisometropia on Other Visual Functions

Ocular dominance [130] is unusually asymmetric in anisometropia, and this imbalance is reduced (improved) after adaptation to a spectacle correction [131, 132]. Vincent et al. found highly symmetrical changes in corneal topography in the fellow eyes of myopic anisometropes after 10 min of reading [133]. Yurdakul et al. found that anisometropia is associated with an increased risk of developing consecutive exotropia after surgery for esotropia [134].

Studies indicate that anisometropia is associated with vergence instability [135] and fixation instability [135, 136], which is strongly correlated with impaired stereopsis [136]. It is believed that anisometropia creates a discordant binocular experience, disrupting ocular motor development [135, 136]. The children with the least instability were those with orthotropic anisometropia without amblyopia, with the presence of strabismus and amblyopia, each associated with increased instability [135]. Chen et al. found evidence of motion misperception, attributed to interocular delay, in anisomyopia [137].

Lee et al. investigated the relationship between anisometropia and horizontal strabismus in a large population of children over 5 years of age and adults of all ages in Korea [138]. Both exodeviations and esodeviations were significantly associated with anisometropia. Other authors have confirmed that anisometropia is associated with an increased risk of strabismus [9, 15, 37], especially microtropia [3, 106]. On average, visual acuity for strabismic anisometropes is 2.5 times worse than for non-strabismic cases with similar anisometropia [94]. Meng et al. found that in Chinese teenagers with myopia and intermittent exotropia, anisometropia was present in 22% of participants [139].

Monovision is a solution to presbyopia where one eye is corrected for distance vision and the other for near [130]. To reduce problems of differential prismatic effects and aniseikonia [3], this approach is usually confined to contact lenses [130] and refractive surgery [140]. A review found the success rate of monovision to be 59–67% [130]. Pollard et al. noted that patients with a history of strabismus or problematic heterophoria are at risk of developing strabismus from monovision, and in such cases, they advocated keeping the induced anisometropia to low levels, such as 1.25–1.50 D [141]. Rosenblatt et al. investigated patients ≥60 years of age, some of whom had undergone cataract surgery to provide pseudophakic monovision [142]. Unexpectedly, pseudophakic monovision did not impact the risk of falls, although pseudophakic single vision was associated with an increased risk of falls compared with patients who had not received cataract surgery. A systematic review of surgically induced anisometropia, typically to create monovision, found that this is well-tolerated but recommended that the induced monovision be kept below 1 D [143].

Cao et al. compared a group of school children with myopic anisometropia with a control group, using a battery of tests of binocular coordination and accommodation [144]. Compared with controls, the children with anisometropia had poorer accommodative amplitude, accommodative facility, vergence facility and symptoms, but better positive relative accommodation. In another control group study, Tang et al. found that all forms of anisometropia (myopia, hyperopic and astigmatic) demonstrated varying degrees of impairment to binocular fusion and stereopsis, with the most pronounced deficits in hyperopic anisometropia [44]. The impact of anisometropia on binocularity may explain why the presence of anisometropia worsens the prognosis for the correction of small-angle esotropia with prisms [145].

### Conditions that Have Been Linked with Anisometropia

Straatsma et al. [146] described an association between unilateral myelinated nerve fibres and ipsilateral myopia causing anisometropia (Straatsma syndrome). Despite the myelination, approximately one-third of cases respond to amblyopia treatment [147].

Nasolacrimal duct obstruction (NLDO) occurs in 5–15% of full-term newborns and spontaneously resolves in 90% of cases in the first 12–18 months of life [148]. Anisometropia occurs at a greater rate in unilateral NLDO patients compared with bilateral NLDO [42, 149], and occurs more commonly in children with NLDO than in the general paediatric population [148, 150], but may develop >60 days after birth [151]. Anisometropia has also been linked with foetal alcohol syndrome [152], developmental coordination disorder [153], Down syndrome [154], congenital ptosis [155, 156], syndromic [32] and non-syndromic [157] craniosynostosis, cardio-facio-cutaneous syndrome from BRAF mutations [158] and diabetes of >15 years duration [159].

Two studies found a slightly higher degree of anisometropia in migraine sufferers than in controls, but neither investigation assessed whether this was while uncorrected [160, 161]. Suzuki and Kiyosawa compared a group of adults with visual snow syndrome and an age-matched control group [162]. The authors found the widely recognised [163, 164] association between visual snow syndrome and migraine, but also found higher mean anisometropia in the visual snow group. Anisometropia was found to be higher in people with autism than in controls [165].

Several [15, 166–168], but not all [169] studies have found a link between prematurity and anisometropia, with the strongest association in children with a history of retinopathy of prematurity severe enough to require laser treatment [15, 166]. Chen et al. found no statistically significant association between symptomatic dry eye and anisometropia in Chinese children aged 12–18 years [170].

As noted above, anisometropia is associated with an increased risk of strabismus [9, 15, 37], especially microtropia [3, 106]. Khorrami-Nejad et al. found anisometropia in 12.9% of patients with Duane syndrome and in 37.6% of patients with Duane syndrome and amblyopia [17].

Jiang et al. investigated clinical and genetic features of cases of unilateral high myopia that had no obvious association with ocular or systemic disease [171]. Genetic defects were identified in about one quarter of these cases, and the authors advocated careful examination of the peripheral retina and genetic screening in such cases.

## Discussion

### Summary of Key Findings

Various diagnostic criteria have been used for anisometropia (Table 2). The most commonly used criterion is SER ≥ 1.00 D, with some studies adding a criterion for astigmatism. Prevalence varies in different regions and with race/ethnicity and age. The risk of anisometropia rises with increasing refractive error [13, 172]. In East and South-East Asian populations, the high prevalence of myopia and rapid myopisation during childhood means that anisometropia is mostly associated with myopia and anisometropia increases in prevalence and magnitude during childhood.

In populations with a relatively low prevalence of myopia, anisometropia continues to be predominantly associated with hyperopia, and is typically stable between the ages of 20–40 years, with ~11–13% of people exhibiting anisometropia ≥1.00 D [13]. There is some evidence that anisometropia has a higher prevalence in Hispanic Caucasians than non-Hispanic Caucasian children [173]. Worldwide, the increasing prevalence of myopia is likely to result in a merging of the distinction between East and South-East Asian and other populations, reflecting a continuum related to the prevalence of myopia in each country.

In summary, the changing epidemiology of refractive errors in many countries means that anisometropia may no longer result predominantly from a failure of emmetropisation to reduce congenital hyperopic anisometropia in young children, but rather may now be a consequence of the development of myopia, especially high myopia, in school-children and adolescents.

The distinction that is drawn in this work between populations from East and South-East Asia and elsewhere contrasts with the review by Barrett et al., which concluded that “there is limited evidence that racial or ethnic differences exert a major influence on anisometropia prevalence” [13]. However, Barrett et al. noted relatively few studies of Asian populations at the time of their review: only five studies from East and South-East Asia are included in Barrett et al.’s Table 1 compared with 17 in Table 3 of the present review.

There are likely two reasons why uncorrected myopic anisometropia is less likely to cause amblyopia than hypermetropic anisometropia. First, the later onset of myopic anisometropia in many cases, and second, because with lower levels of myopia, it is likely that each eye will be used at one fixation distance. High myopic anisometropia can lead to amblyopia, and if high myopia is found in a young child, then syndromic forms of myopia and genetic causes should be considered [174]. The often unilateral nature of anisometropia probably explains why it is less commonly detected than more symmetrical refractive errors [175]. This highlights the need for vision screening programmes and/or routine professional eye care.

Concerning myopia control interventions for anisometropia due to unilateral myopia, the literature presents clinicians with a dilemma. If the primary goal is to reduce anisometropia, then prescribing a myopia control intervention to the myopic eye only is recommended, as this is likely to reduce anisometropia over time. However, an underlying reason for this reduction in anisometropia over time is that the onset of myopia in one eye often heralds the development of myopia in the initially unaffected eye. Owing to the importance of myopia prevention [176], it could be argued that the primary goal should be to reduce the child’s myopia progression in both eyes, even in cases where only one eye is myopic.

The decision whether to prioritise the treatment of anisometropia or to initiate myopia control in both eyes likely depends on the presentation of the case. Myopia control in both eyes is probably indicated if there is evidence of pre-myopia [65], axial elongation or departure from normal axial length growth curves in the better eye. Another factor to consider is that myopia control interventions are generally not licensed for pre-myopia, so consent would be required for off-label product use.

Most studies of bilateral OK for myopic anisometropia report a reduction in anisometropia over time. This may be related to the findings of a recent review of myopia control interventions for high myopia [177]. This study found that OK is more effective than peripheral plus spectacle lenses for reducing axial elongation in high myopia. It seems likely that the amount of corneal deformation required for OK to correct high degrees of myopia will also create increased peripheral myopic defocus and/or greater contrast reduction of the retinal image. This may explain why OK appears to be more effective than optical myopia control interventions in high myopia, and bilateral OK seems to have a relatively greater effect in the more myopic eye, thus reducing myopic anisometropia.

Concerning the effect of anisometropia on visual functions, anisometropia, especially higher degrees, impairs stereoacuity, creates atypical ocular dominance and increases the risk of strabismus, especially microtropia, and amblyopia. Indeed, several developmental and other conditions were noted above to be associated with an increased prevalence of anisometropia.

Anisometropia is associated with aniseikonia and differential prismatic effects, which increase the risk of spectacle non-tolerance [3]. A historical hypothesis, based on an interpretation of Knapp’s Law, which predicted that aniseikonia is minimised by contact lenses in cases of refractive anisometropia and by spectacles in axial anisometropia, has been disproven [99]. For all cases of anisometropia, aniseikonia is likely to be reduced by wearing contact lenses or by refractive surgery, which also avoids differential prismatic effects [3].

### The Difficulty of Providing Cut-offs

Trainee eye care practitioners often ask three simple questions about cut-offs for when anisometropia is likely to be problematic:

1. In children, what level of anisometropia is likely to cause amblyopia?
2. In older children and adults, when should anisometropia be corrected?
3. When is aniseikonia likely to cause problems?

To determine the “received wisdom”, Table 4 provides recommendations on these questions from several textbooks. Dobson et al. found that non-astigmatic hyperopic children with ≥1.00 D of anisometropia showed significantly increased interocular differences in visual acuity [6]. Concerning symptoms, Millodot states that uncorrected anisometropia of a low degree may cause eyestrain or diplopia, but higher levels rarely cause symptoms because one of the images is typically suppressed or there is amblyopia [1]. This suggests that there is a non-linear relationship between the magnitude of anisometropia and the likelihood of symptoms, which may explain inconsistencies in the literature. In addition to this point, the divergent views in Table 4 are explained, at least in part, by the fact that individuals differ considerably in the amount of anisometropia and aniseikonia that can be tolerated [3].

**Table 4:** Recommendations from textbooks.

Book authors/editors Book title	Year Edition	When does anisometropia require correction?	When is anisometropia likely to cause amblyopia?	When is aniseikonia problematic?
Bennett and Rabbetts [113] Clinical visual optics	2007 4e	2.00–3.00 D (presbyopes)	No value given	No value given
Keirl and Christie [105] Clinical optics and refraction	2007	No value given	No value given	0.25% in some cases
Rosenfield and Logan [109] Optometry	2009 2e	May cause problems ≥1.00 D	>1.50 D after age 3 years	No value given
Rowe [106] Clinical orthoptics	2012 3e	>1.00 D	No value given	>3%
Elliott [261] Clinical procedures in primary eye care	2021 5e	No value given	No value given	No value given
Millodot [1] Dictionary of optometry	2018 8e	No value given	No value given	No value given
Scheiman and Wick [262] Clinical management of binocular vision	2020 5e	1.00 D (0.50 D if convergence insufficiency)	>1.25 D	>1.25%
Evans [3] Pickwell’s binocular vision anomalies	2022 6e	>1.00–2.00 D	>1.50 D	>2%
Stokkerman and Day [108] Aniseikonia	2025 1e	No value given	No value given	>1–3%

*e* edition.

Krarup et al. found wide inter-individual variation in the amount of aniseikonia that normal adults can tolerate without impairing stereopsis [115]. One in five individuals had impaired stereopsis with induced aniseikonia of as little as 1%, while over half could tolerate >10% aniseikonia without loss of stereopsis. This study only measured the immediate effect of aniseikonia and, therefore, may have underestimated inter-individual variability. Variations between individuals in sensory adaptations and tolerance probably explain why different people vary so much in their ability to adapt to spectacle correction of anisometropia. This, in turn, is likely to underlie the wide differences between prescribing patterns of different eye care practitioners [178].

### Limitations and Recommendations for Research

A limitation of this review is that studies are included that used different criteria for defining anisometropia. The alternative approach, to only include studies that used the most common definition, would have required excluding a large portion of the literature. A limitation of the review on prevalence is that this only considers the most recent 5 years. This decision was taken in view of the increasing prevalence of myopia, and therefore anisometropia, in many countries. This means that more historical data are unlikely to reflect the current situation.

Further research, both prospective randomised controlled trials (RCTs) and observational studies, would be useful to investigate whether the early correction of anisometropia in children with contact lenses improves outcomes in terms of binocularity and prevention of amblyopia. It would also be interesting to investigate whether the correction of adults with longstanding anisometropia results in improvements in binocularity and visual acuity.

Concerning research to evaluate myopia control interventions, although RCTs are essential, they are not sufficient. A limitation of RCTs is that participant exclusion criteria may limit the relevance of the findings to clinical practice, for example, by excluding participants with anisometropia. Trials with broader inclusion criteria are advocated [179] in addition to observational studies of “real-world data” [180] from clinical populations [181, 182]. Finally, since both of an individual’s eyes will have an identical genetic background and are likely to have similar environmental experiences, the development of markedly different refractive errors in each eye in individuals with anisometropia may provide insights into the aetiology of refractive errors [46].

## Data Availability

Outputs of literature searches can be provided upon reasonable requests to the authors.
